# Novel doses of sacubitril/valsartan in patients unable to tolerate traditional therapy: Effects on N‐terminal pro B‐type natriuretic peptide levels

**DOI:** 10.1002/clc.23509

**Published:** 2020-12-05

**Authors:** Amitabh C. Pandey, Derek Jer, Ruth S. Kuo, David H. Yoo, Antonio Christophy, Rajeev C. Mohan, Ajay V. Srivastava, James Thomas Heywood

**Affiliations:** ^1^ Division of Cardiology Scripps Prebys Cardiovascular Institute, Scripps Clinic La Jolla California USA; ^2^ Scripps Research Translational Institute The Scripps Research Institute La Jolla California USA; ^3^ Department of Pharmacy Scripps Clinic La Jolla California USA; ^4^ Section of Advanced Heart Failure and Mechanical Circulatory Support, Division of Cardiology Scripps Prebys Cardiovascular Institute, Scripps Clinic La Jolla California USA

**Keywords:** angiotensin receptor blocker and neprilysin inhibitor, heart failure with reduced ejection fraction, loop diuretics, N‐terminal pro brain natriuretic peptide

## Abstract

**Background:**

Widespread use of angiotensin receptor blocker and neprilysin inhibitor (ARNI) remains low, and many patients are unable to tolerate the medication due to hypotension at the currently recommended starting dose.

**Hypothesis:**

The aim of this study is to assess if lower than standard doses of ARNI, sacubitril/valsartan (S/V), significantly reduces NT‐proBNP and leads to any change in diuretic dose, serum potassium, or creatinine.

**Methods:**

In a retrospective study of 278 patients who were started on a low dose S/V at a single medical center, 45 patients were selected for the study cohort. Patients were subcategorized to Group 1 (*n* = 10): very low dose S/V (half a tab of 24/26 mg BID), Group 2 (*n* = 10): very low dose titrated to low dose S/V, and Group 3 (*n* = 25): low dose S/V (24/26 mg BID). NT‐proBNP, diuretic dose, serum potassium, and creatinine were compared before and after initiation of S/V.

**Results:**

Among all groups, there was a significant reduction in NT‐proBNP level (Group 1: p < .01, Group 2: p < .01, and Group 3: p < .001). In addition, there was a significant reduction in diuretic dose across all groups combined (furosemide 53 mg/day vs. 73 mg/day; p = .03), with 17.8% (8/45) patients being able to discontinue their diuretic completely. There was no significant change in potassium or creatinine.

**Conclusions:**

Lower than standard dose of S/V significantly reduces NT‐proBNP and diuretic requirement without change in potassium or creatinine, which provides hope that patients who cannot tolerate standard doses of S/V due to hypotension may be able to receive the benefits of S/V therapy.

## 1 INTRODUCTION

Heart failure (HF) is one of the most detrimental and costly disease processes in medicine. Its influence is far reaching, affecting patient morbidity and mortality as well as overall healthcare costs. It is estimated that over 6.5 million adults suffer from HF in the United States and nearly 40 million adults affected worldwide.^1^, ^2^ Recently, the Prospective Comparison of ARNI with ACEI to Determine Impact on Global Mortality and Morbidity in Heart Failure (PARADIGM‐HF) trial demonstrated significant promise for the future of pharmaceutical therapy in treatment of HF patients.^3^ Sacubitril/valsartan (S/V) has been shown to be efficacious in many trials that have been completed or are ongoing.^3^, ^4^, ^5^, ^6^, ^7^ The ability to act not only on RAAS system and reduce afterload as well as capitalize on the volume titration properties of NPs seems, in hindsight, to be an ideal combination. Furthermore, it is well established that NPs play a pivotal role in the process of natriuresis—and in turn diuresis—ultimately becoming a pivotal regulator of cardiovascular homeostasis through their paracrine and endocrine actions.^4^, ^8^, ^9^

In the PARADIGM‐HF trial, a 20% reduction in cardiovascular death or hospitalization for HF in the PARADIGM‐HF led to the approval of S/V from the US Food and Drug Administration (FDA) as well as the European Commission in July 2015 and November 2015, respectively.^3^ Furthermore, S/V earned designation as the first line therapy in a focused update of the ACC/AHA guidelines for the treatment of HF as well as the European HF clinical practice guidelines.^10^, ^11^, ^12^, ^13^ After a run‐in phase that ensured all patients were able to tolerate enalapril 10 mg twice daily, patients were switched to S/V 49/51 mg twice daily and finally S/V to a goal dose of 97/103 mg twice daily.^6^ Ultimately, the FDA approved three doses: a 23/24 mg (low dose), 49/51, and 97/103 mg (high dose). Subsequent to PARADIGM‐ HF, many post‐hoc analysis were conducted to investigate hypotension with S/V. While patients were more likely to experience hypotension in the run‐in phase of PARADIGM‐HF with S/V therapy as compared to ACEI, these patients still derived benefits from S/V therapy.^14^, ^15^ Although the dose of S/V used was still among the standard dosing.^15^ Subsequent “real world” studies also show a significant increase in hypotensive episodes with S/V as compared with ACEI.^16^ Thus, despite estimates of profound benefit to HF patients, in current clinical practice, many patients cannot tolerate high doses of S/V, or even the smallest approved dose of 24/26 mg twice daily due to several factors including hypotension.^10^, ^17^, ^18^ This remains even more so in older patients.^14^ It remains unclear if lower doses of S/V are associated with reduction in NT‐proBNP levels. In the current study, we aimed to investigate if S/V doses below the lowest approved 24/26 mg tablet correlate with reduction in NT‐proBNP and associated beneficial effects in patients with HFrEF.

## 2 METHODS

### 2.1 Study design

Using a retrospective observational analysis at a single medical center, NT‐proBNP levels were compared before and during S/V therapy, as a surrogate indicator for S/V therapy efficacy. Electronic medical record (EMR) data were queried for patients on S/V therapy between October 2015 and January 2018. Data were collected from EMR, Medicare Severity Diagnosis Related Groups (MS‐DRG) and IBM COGNOS, v.10 (Armank, NY). All patients starting S/V at either very low dose (half of a 24/26 mg tablet twice a day) (VLDS/V), or low dose (24/26 mg tablet BID) (LDS/V) were included. Patients were excluded if there were no NT‐proBNPs obtained before or after implementation of S/V therapy, or if patients were lost to follow up in clinic. Patients were divided into three groups based on S/V dosing. Group 1 patients received VLDS/V throughout the evaluation period, Group 2 patients received VLDS/V and were titrated to LDS/V, and Group 3 patients received LDS/V throughout the evaluation period. Patients were evaluated for a minimum of 2 weeks, with no maximum monitoring periods stipulated. The study protocol received an approval of the Scripps Clinic Institutional Review Board.

### 2.2 Primary and secondary endpoints

Primary endpoint was the mean change in NT‐proBNP values between the baseline and last value at a follow‐up. Baseline NT‐proBNP values were obtained within 30 days prior to the S/V therapy initiation. Duration of therapy referred to the time between initiation of S/V and a follow‐up serial NT‐proBNP level. The secondary endpoints included mean changes in loop diuretic dose, serum potassium, and creatinine. Loop diuretic doses were converted to furosemide equivalents (1 mg bumetanide = 20 mg torsemide = 80 mg furosemide for oral diuretics, and 1 mg bumetanide = 20 mg torsemide = 40 mg furosemide) for standardization of pharmaceutical therapy, as previously described.^19^

### 2.3 Statistical analysis

All statistical analyses were performed using the Wilcoxon Rank‐Sum test using GraphPad Prism software, Version (La Jolla, CA). Analysis for NT‐proBNP level was performed between the baseline and final assessment after therapy. Difference in diuretic dosing was assessed for statistical significance as well with the Wilcoxon Rank‐Sum test. Significance was assessed by p ≤ .05. All analyses were conducted at the two‐tailed level, and the significance was set at α ≤ .05.

## 3 RESULTS

**FIGURE 1:**
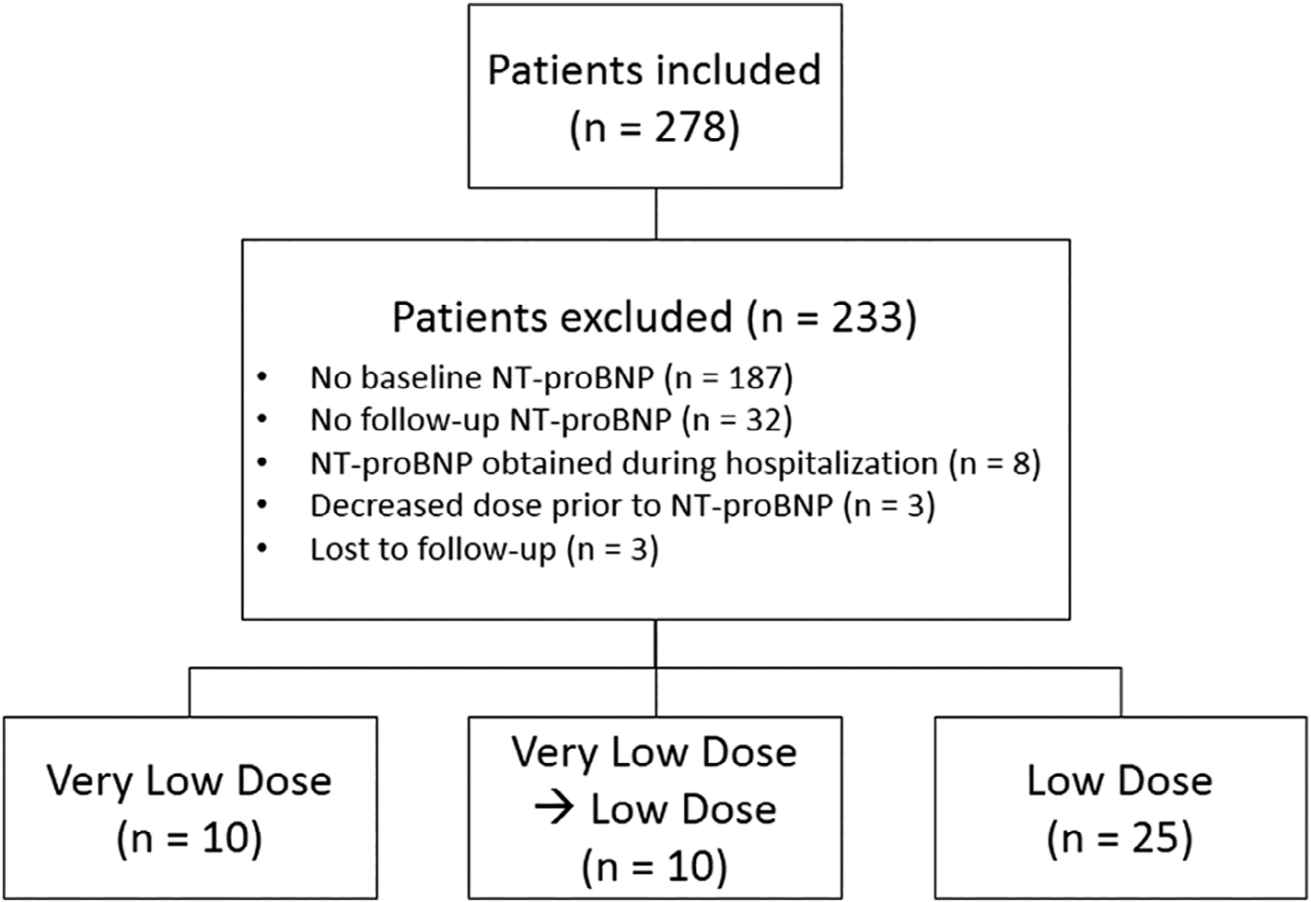
Study design to evaluate very low dose sacubitril/valsartan and changes in NT‐proBNP expression. Study design demonstrating all patients screen and stepwise exclusion whereby final study cohorts were derived. Three groups were utilized in the study, based on dosing of sacubitril/valsartan administered. Group 1 patients received very low dose throughout out the evaluation period, Group 2 patients received very low dose and were ultimately titrated to low dose, and Group 3 patients received only low dose throughout the evaluation period

A total of 278 patients were screened who had started S/V, with 45 patients forming the study cohort (Figure 1). Patients were divided into three groups: Group 1, *n* = 10 (VLDS/V); Group 2, *n* = 10 (VLDS/V to LDS/V), and Group 3 *n* = 25 (LDS/V) (Figure 1). The average age, across all groups, was 69 ± 15 years, with 84% of the participants being male (Table 1). Fifty‐five percentage of the patients had an ischemic cardiomyopathy (ICM), and the average left ventricular ejection fraction (EF) across all groups was 28 ± 8.5%. Minimal variability in demographics was observed among three groups (Table 1). The average systolic blood pressure (SBP) across all groups at the initiation of S/V was 107.6 ± 12 mm Hg, with lower observed SBP for Groups 1 and 2 as compared to Group 3 (Table 1). Average days from baseline NT‐proBNP and initiation of S/V was 5.9 + 8.1 days, and the average duration of therapy was 167.2 ± 148.8 days.

**TABLE 1:** Demographic characteristics of study populations

Characteristics	All groups (*n* = 45)	Group 1 (*n* = 10)	Group 2 (*n* = 10)	Group 3 (*n* = 25)	p value
Age (years)	69.2 ± 15.3	68.7 ± 11.5	66.7 ± 18.1	70.4 ± 15.9	NS
Sex (female)	45 (7)	10 (0)	10 (3)	25 (4)	NS
SBP pre (mm Hg)	107.6 ± 9.5	103.4 ± 7.8	102.2 ± 9.6	111.52 ± 7.8	NS
SBP post (mm Hg)	103.3 ± 16.9	98 ± 15.9	100.9 ± 14.1	106.4 ± 15.9	NS
EF (%)	28 ± 8.5	29 ± 8.5	26 ± 9.0	28 ± 8.3	NS
ICM (% of pt)	56%	50%	50%	60%	
DM (% of pt)	40%	30%	60%	36%	
Days from NT‐proBNP and therapy	5.9 ± 8.1	3.9 ± 6.4	4.8 ± 5.6	7.1 ± 9.4	NS
Duration of therapy (days)	167.2 ± 148.8	112.7 ± 108.2	149.4 ± 98.5	196.2 ± 174.2	NS
Number of hospitalizations (hospitalizations for HF)	11 (3)	2 (0)	2 (1)	7 (2)	NS
Pre‐therapy potassium level (mEq/L)	4.3 ± 0.5	4.1 ± 0.5	4.1 ± 0.5	4.4 ± 0.5	
Post‐therapy K level (mEq/L)	4.5 ± 0.3	4.5 ± 0.4	4.4 ± 0.4	4.6 ± 0.3	NS
Pre‐therapy Cr level (mg/dl)	1.19 ± 0.4	1.14 + 0.3	1.29 + 0.3	1.17 ± 0.4	
Post‐therapy Cr level (mg/dl)	1.17 ± 0.5	1.18 + 0.3	1.17 + 0.4	1.16 ± 0.6	NS
Baseline diuretic dose (mg of furosemide/day)	73 ± 12.6	85 ± 21.4	63 ± 14.1	72 ± 19.3	
Post‐therapy diuretic dose (mg of furosemide/day)	53 ± 13.7*	87 ± 23.7	41 ± 16.4	42 ± 12.1*	p < .05
Baseline beta blocker dose (mg of coreg/BID)	18.9 + 2.9	10.9 + 3.3	21.6 + 9.6	21 + 3.4	
Post‐therapy beta blocker dose (mg of coreg/BID)	18.1 + 2.8	9.7 + 2.9	21.6 + 9.2	21 + 3.1	NS
Baseline MRA dose (mg/day)	14 + 2.9	24.2 + 2.9	8.75 + 9.6	11.9 + 3.4	
Post‐therapy MRA dose (mg/day)	12.3 + 2.8	11.8 + 2.9	13.8 + 9.2	11.9 + 3.1	NS

*Notes:* Patient characteristics at baseline and after therapy with sacubitril/valsartan. Data for the overall cohort, very low dose, titration, and low dose cohorts for baseline characteristics as well as changes in laboratory assessments of patients. Data are represented as mean ± SD, where appropriate, *, **, *** representing p < .05, .01, and <.001, respectively. Significance for pre/post S/V compared, where appropriate. No significant differences were noted among three groups, except diuretic dosing. Diuretic dosing had significant differences between groups as well as pre/post SV therapy. Diuretic dose is represented as milligrams of furosemide equivalents (furosemide 40 mg = 1 mg bumetanide = 20 mg torsemide).

Abbreviations: EF, ejection fraction; HF, heart failure; ICM, ischemic cardiomyopathy; SBP, systolic blood pressure.

### 3.1 Changes in NT‐proBNP levels

**FIGURE 2:**
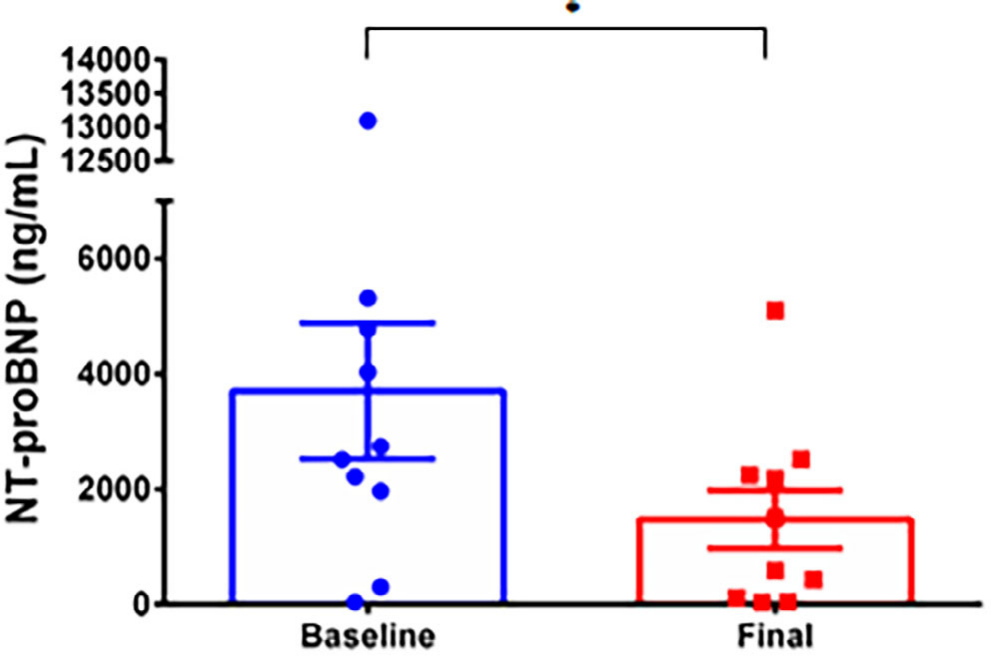
Effect of very low dose sacubitril/valsartan on NT‐proBNP. Baseline and final measurements of NT‐proBNP were obtained for Group 1 (started and maintained on very low dose 12/13 mg BID of sacubitril/valsartan [S/V]). Significant reduction in NT‐proBNP is demonstrated even with very low dose S/V. Data are represented as mean ± SE, ** representing p < .01

**FIGURE 3:**
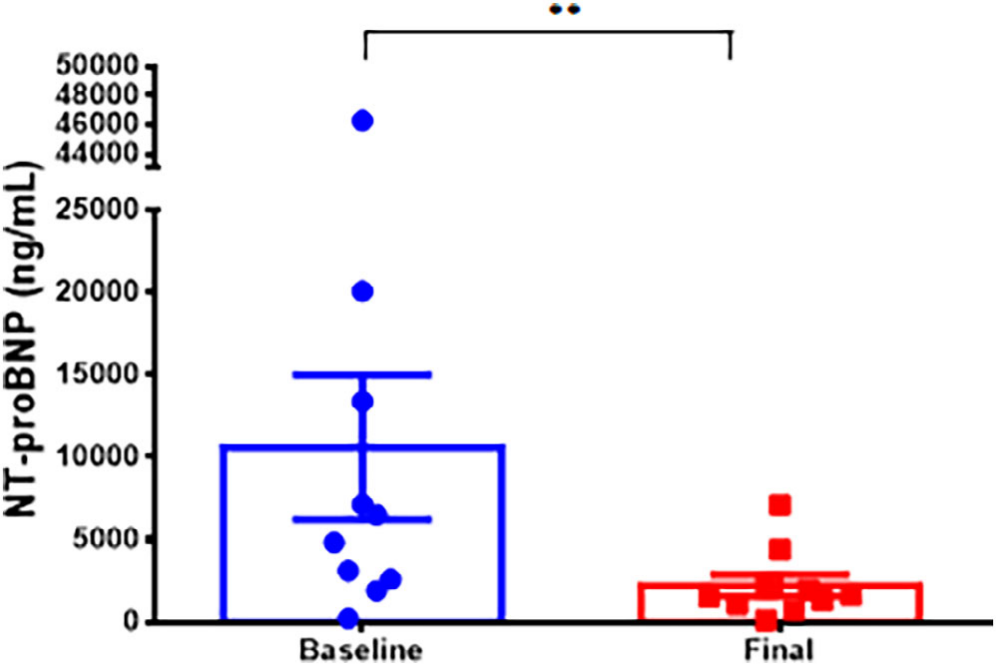
Effect of dose titration of sacubitril/valsartan on NT‐proBNP expression. Baseline and final measurements of NT‐proBNP were obtained for Group 2 (started on very low dose 12/13 mg BID of sacubitril/valsartan [S/V] and titrated during study period to low dose S/V of 24/26 mg BID). Significant reduction in NT‐proBNP is demonstrated even after starting at the very low dose. Patients of this group were able to tolerate increase in S/V to the low dose, without discontinuation of therapy. Data are represented as mean ± SE, ** representing p < .01

**FIGURE 4:**
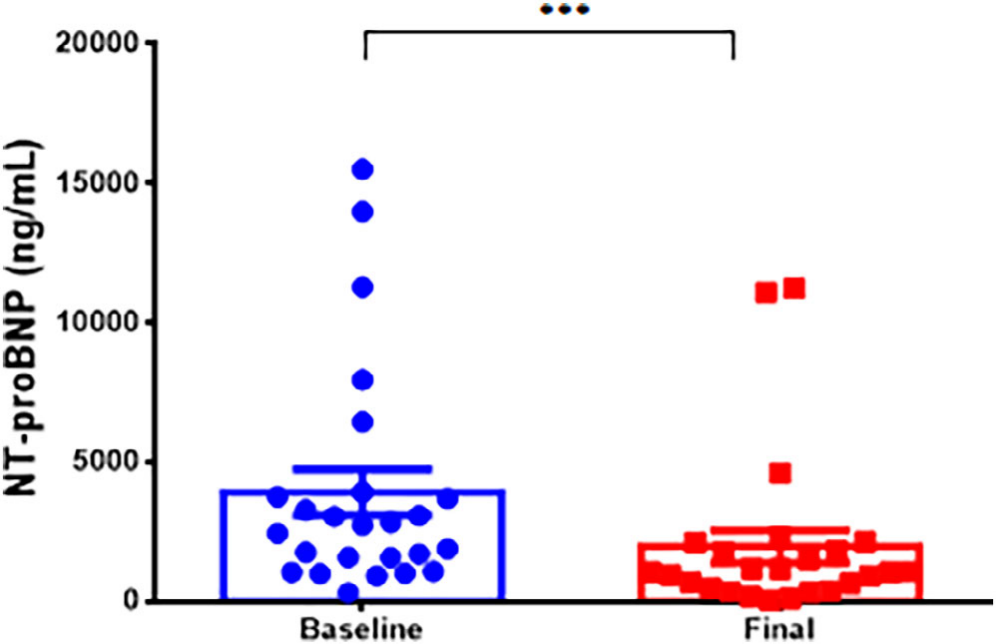
Effect of low dose sacubitril/valsartan on NT‐proBNP expression. Baseline and final measurements of NT‐proBNP were obtained for Group 3 (low dose group started and maintained on 24/26 mg BID of sacubitril/valsartan). Data are represented as mean ± SE, *** representing p < .001

S/V therapy resulted in a significant decrease in NT‐proBNP levels in all groups. Group 1 demonstrated a significant reduction in NT‐proBNP levels from 3703 to 1478 ng/ml (p < .01) (Figure 2). NT‐proBNP decreased from 10 629 to 2232 ng/ml (p < .01) in Group 2 (Figure 3) and from 3916 to 1965 ng/ml (p < .001) in Group 3 (Figure 4). Patients in Group 3 had the smallest reduction in NT‐proBNP levels (50%), while patients in Group 2 had the largest reduction (79%) in NT‐proBNP. The amount of decrease in NT‐proBNP levels was also assessed. Across all dosing groups, patients were assessed for reduction in NT‐proBNP levels below 1000 ng/ml. A total of 13 of 45 patients met this threshold (Group 1: 3 patients, Group 2: 1 patient, and Group 3: 9 patients).

### 3.2 Changes in administration of loop diuretics

Of the total cohort, 69% (31/45) received a loop diuretic therapy prior to S/V (8 in Group 1, 7 in Group 2, and 17 in Group 3). An average daily dose of loop diuretics decreased significantly after initiation of S/V (53 mg/day vs. 73 mg/day; (p = .03). Statistical significance was driven by Group 2 and 3 as there was no significant change in daily diuretic dosing in Group 1 (Table 1). Furthermore, 18% (8/45) were able to discontinue their diuretic therapy completely (none in Group 1, 3 in Group 2, and 5 in Group 3).

### 3.3 Changes in serum potassium and creatinine

Overall, creatinine did not change across all groups. There was a trend for improvement in serum creatinine levels although Group 1 had non‐significant increase in creatinine (1.14–1.18 mg/dl) (Table 1). There was a non‐significant trend towards increase in potassium.

## 4 DISCUSSION

This study demonstrates an improvement in NT‐proBNP in patients who are unable to tolerate the minimum recommended initiation dose of S/V. Additionally, significant reduction in diuretic dosing after initiation of S/V is observed. Despite rapid Class I recommendation status in the AHA/ACC treatment guidelines, previous studies have shown that only 12% of medical practice patients who were indicated for S/V were receiving therapy.^20^ Further, adoption of S/V therapy has been slow in hospitalized patients, with only 2.3% of patients receiving S/V therapy on hospital discharge, despite recent trials.^7^, ^10^

It is not surprising that older patients would either be on LDS/V or not receive the agent at all.^15^ Patients in the current study were not only considerably older than those in PARADIGM‐HF (69 vs. 61 years, respectively), but also had lower SBP as compared to both the titrated and non‐titrated cohorts in PARADIGM‐HF (107.6 vs. 120.7 vs. 121.9 mm Hg, respectively). Of note, patients in Group 1 with an average SBP 103.4 mm Hg prior to S/V initiation were able to tolerate VLDS/V despite average SBP decreasing to 98 mm Hg.

NT‐proBNP has proven to be a valuable prognostic biomarker in HF. Zile et al demonstrated that patients with NT‐proBNP levels <1000 in the PARADIGM‐HF study had a significantly better prognosis compared to >1000 and had a similar risk of cardiovascular death or hospitalization as those who started with similar lower NT‐proBNP levels.^21^ Remarkably, even in Group 1 of the present study, patients were able to achieve reduction in NT‐proBNP by initiating LDS/V, who would have otherwise not been started on S/V. Despite higher initial NT‐proBNP levels, and lower S/V initiating dose, there was still a significant decline in some of our patient cohort achieving levels below 1000 ng/ml, across all dosing groups in the present study. Interestingly, when comparing the VLDS/V and LDS/V, about 30% of the patients were able to meet this reduction threshold.

In addition, patients were able to achieve reduction in diuretic dosing on S/V therapy, with some being able to discontinue all diuretic therapy. Reduction in diuretic therapy may be tied to the natriuresis resulting from increased circulating NP levels.^22^ Given that no patient in Group 1 on VLDS/V therapy was able to discontinue diuretic therapy, it seems that while there is significant reduction in NT‐proBNP levels with VLDS/V, concurrent diuretic therapy is still needed to achieve euvolemic state. However, there were patients both in Group 2 who were bridged from VLDS/V to LDS/V and in Group 3 on LDS/V, who were able to discontinue all diuretics while on S/V therapy.

As yet there is minimal data on the efficacy of S/V at doses below the target dose of PARADIGM‐HF. One study reported that patients in PRADIGM‐HF who were down‐titrated to reduced doses of S/V did better than those on reduced doses of enalapril, although the reduction was from high dose to moderate dose S/V therapy.^15^ Furthermore, patients in PARADIGM‐HF who suffered from hypotension, benefited from S/V therapy similar to those without hypotension.^14^ Again, these patients had standard and often at least moderate dose therapy S/V. The present study shows indications of similar results with lower doses‐ potentially allowing more patients to gain the benefits of ARNI therapy. Realistically, in clinical practice, these medications are often discontinued despite the benefit that may be achieved. This study demonstrates that reductions in NT‐proBNP can be achieved even at a VLDS/V thereby justifying the rationale for initiation of this drug at a very low dose in those who may not otherwise be started on the drug.

Our study has several limitations especially since it is a small single‐center, retrospective study and clinical outcomes were not assessed. The purpose of the study was to determine if initiation and continuation of lower than recommended doses of S/V produced a salutary effect on NT‐proBNP in a cohort with low systemic blood pressure.

## 5 CONCLUSION

S/V is a hallmark therapy that reduces mortality and morbidity in HFrEF patients as evidenced in PARADIGM‐HF trial. Usage of S/V in clinical practice remains low, in part limited by hypotension. This study supports the concept that patients who may not have received S/V due to low blood pressure can tolerate VLDS/V and LDS/V, which results in a significant reduction of NT‐proBNP without any significant change in serum potassium or creatinine. Moreover, in patients who were able to tolerate LDS/V, there was a significant decrease in their diuretic dose and some were able to stop their diuretic completely. While our study demonstrates a significant biomarker response to lower than standard dose of S/V, further investigation is needed to confirm clinical benefit with non‐target doses of S/V.

## DISCLOSURE OF INTERESTS

Amitabh C. Pandey, Derek Jer, Ruth S. Kuo, David H. Yoo, Antonio Christophy, Ajay V. Srivastava, Rajeev C. Mohan have no relevant disclosures; James Thomas Heywood—Speaker and consultant of Novartis.

## Data Availability

The data that support the findings of this study are available on request from the corresponding author. The data are not publicly available due to privacy or ethical restrictions.
